# Monthly Summary

**Published:** 1899-01

**Authors:** 


### Monthly Summary.

NEW METHOD OF DISINFECTION.—Nc sanitary subject has received more attention lately than that of disinfection. Drs. Walther and Schlossmann give the following details of a new method of disinfection: By means of a specially constructed apparatus, a mixture of formaldehyde and glycerine is sprayed into a room which is to be disinfected, until a thick fog results; about 4 pounds of the mixture are needed per 1,000 cubic feet. The room need not be hermetically closed during the operation, as the ordinary circulation of air assists in spreading the disinfectant, and in enabling it to reach remote corners. Three hours' exposure was found sufficient to kill all germs in the rooms experimented on, though the test objects were purposely chosen of the most refactory nature.

For example: Pieces of linen thickly coated with a paste of white of egg and garden soil, dried in an incubator; layers of soil 3 or 4 millimeters thick with potato skins under and above them; potato skins alone. These were placed, open and covered, at various heights in the room, in recesses in the wall, on the floor, under pieces of furniture, in tall glass cylinders, or in shorter cylinders under a laying of wadding, in the pockets of thick winter clothing. Feces were also sterilized by this exposure. Live Guinea pig and rabbits were also found to be freed from bacteria in their skins, their bedding straw, and their excrement.

The authors attribute the very advantageous effect of adding glycerine to the formaldehyde to its hygroscopic character and its power of adhering to and penetrating most of the ordinary porpous materials found about a household. They anticipate that it may be found possible to diminish still further the necessary duration of the period of disinfection, and that their method will become a much more powerful agent than any yet known against the spread of infectious diseases, not only in man, but in the lower animals.—*Prakt. Chem.*

CATAPHORESIS—A WARNING.—The experience of a thousand wise predecessors will not divert folly. We must all get experience for ourselves, and no doubt it is a providential dispensation. But there is no excuse for that presumption which dogmatically arrogates an almost divine right of judgment, which, in the infancy of a new idea, or a novel experience, insists that it has encompassed within its opinion, every possibility of fact or failure.

The cataphoresis fad is not altogether new. It is not one of those fads which are without any merits. It has been successfully applied in clinics—though the value and permanency of results are not fully seen in clinics. It is a fad which requires some special prediliction and experience in a direction wherein few dentists technically excel. It is not a thing to be despised or condemned because of its failures, but rather a thing where failures should inspire to conquest. Some of its advocates have been, perhaps, too assertive in their declarations that they have seen no harm in it, that they have done no harm with it. It is generally impossible to know at once whether or not mischief has been done. But recently facts are coming forth to prove that toxic effects of cocaine upon the pulp have extended beyond that tissue to a dangerous condition.

Dr. M. W. Foster, of Baltimore, reported a case in the *Cosmos*, where a pellet of cotton, saturated with a 30 per cent. solution of cocaine hydrochlorate was placed in the cavity of a tooth, and the usual cataphoric current applied. The dose was repeated. About ten minutes from the last application the patient complained that the fingers of the left hand felt as if they were asleep. The symptoms became so alarming that brandy was hypodermically injected and friction applied; the patient "commenced a peculiar howling sound." The condition lasted from 3.30 p. m., until 8.15 p. m. The rigidity of the body was very marked. The power of speech was not regained until 8.15 p. m., after digitalis had been administered. The throat was very dry for a night and a day; the eyes painful, and for some time the vision was dimmed. This case is, perhaps, more pronounced than any most of us have witnessed; but, within the limited opportunities for observation, a sufficient number of cases have been known to justify extra precaution, especially on the part of those enterprising bluffers who think the possession of electrical apparatus and the merest smatter of knowledge, sufficient stock-in-trade to entitle them to rush in where experts are cautious about treading.—Dominion Dental Journal.

---

TRANSPLANTING—RESETTING.—I have read, with much relish, the various reports lately published in the dental journals on the subject of transplanting, resetting, budding and root pruning of natural teeth, and of the marvelous and invariably satisfactory results attained. How any one can doubt that teeth can be transplated, just like young beets or turnips, and be better and more flourishing than ever, is a marvel to me. That any body, interested in the character of the profession, should look grave when reading the accounts of the root-pruning indulged in on such occasions, and the perfectly satisfactory results attained thereby, only proves how much our profession has yet to learn. The smile I perceived on the countenance of one of our most eminent practitioners, while reading the accounts of the planting of a tooth which had lain dry and dead for weeks in the drawer of a celebrated doctor of dentistry, as related in one of the late numbers of your journals, filled my heart with pity, and I resolved to add my testimony as to the perfect feasibility of such operations.

The fact is, the enterprise of operators who have experimented on this line of dental practice has stopped far short of what can be done.

I have on many occasions inserted into the bleeding cavities, left by extraction of teeth, cows', sheep's or pig' teeth, with perfectly satisfactory result, and my practice has been for many years to keep on hand, for the use of patients with small means, a graduated lot of ivory pegs, shaped like teeth, which can be planted quite successfully. These pegs can be trimmed to match, before the teeth which they are to replace are extracted, and can be run into their place at once—the shape of the root being of no consequence whatever. In cases where

Nature has made a mistake in that harmony which ought to assist in the different parts of the head forming expression, these mistakes can easily be corrected, so far as the teeth are concerned, by giving the pegs such shape an inclination as will produce the desired effect. A bill will be presented in the next session of Congress, in which I propose, for a reasonable remuneration, to plant ferocious-looking fangs into the mouths of the United States troops. These will produce such a fearful expression as will strike terror into the hearts of the painted savages, and will greatly shorten our Indian wars. After the war these fangs may be extracted and deposited at the arsenals for use in the next war, while the peaceful soldier can be planted with meek-looking sheep's, cows', pigs', calves' or goats' teeth, or with pegs of ivory, walrus or hicory, as he may choose, all of which will grow tight at once, and will be infinitely better than their original predecessors. I append two photographs illustrating this process.—DR. A. J. Volck, in *Johnson's Miscellany*.

---

EXTRACT FROM AN ARTICLE UPON "SOME OVERLOOKED SOURCES OF INFECTION."—Uncleanliness in dental surgery need only be mentioned briefly in passing, since all discussion of the subject should be politely suggested, alluding as it does to a profession which consists of scientific men who are already somewhat sensitive upon the question of their relations to the medical profession. It is not my intention however, to direct attention to this desire on the part of some of these gentlemen to be ranked as specialists with the oculist and the aurist and the gynecologist, but rather to the fact that in dental offices in general, or, to put it more mildly, in a very large proportion of dental offices throughout the country, there is a marked negligence in the proper care and sterilization of instruments and appliances. Numerous instances of infection from this source have been traced, and many cases of necrosis and subperiosteal abscesses, to say nothing of constitutional infection, have been among the deplorable results of unclean dentistry. That such results are inexcusable goes without saying, and the mere invitation to extreme care in their work, given on the part of the medical profession, should be all sufficient to cause dental surgeons everywhere to adopt through and complete aseptic methods for the most trivial operations. If not, then let it be made a penal offense to thrust into the soft and highly absorbent tissues of the human mouth forceps that have recently been polluted by the festering jaws of the scorbutic, the cankerous or the syphilitic, and only cleaned by a more or less careful wiping with a piece of absorbent cotten. A careful and extensive inquiry reveals the fact that in the great majority of dental offices in California, except in cases of suspected disease, instruments are seldom surgically cleaned, either before or after operations. No surgeon nowadays would lance a boil, or cut for a sliver, without being sure of his bistoury, and no dental surgeon should pull a tooth or incise a gum without equal care.—M. L. Dyer, in *Western Dental Journal.*

---

Tincture of Iodine for the Removal of Deposits.—Apply the dilute tincture freely to teeth and gums, which will constringe puffy gums, drawing them away from about the teeth and outline deposits, which might otherwise escape detection. It loosens deposits upon the teeth, insinuating itself into minute crevices and rough areas of the crowns. Follow the iodine with ammonia, which forms a colorless solution, leaving the teeth much lighter in color. Clean and polish the surfaces.—Dr. Register, *International.*

---

Bleeding Gums.—To obviate bleeding of the gums in crown- and bridge-work, apply a 25 per cent. solution pyrozone. This acts as a styptic, one or two applications rendering the gum perfectly dry for from ten to fifteen minutes.—Conrad E. Wittlaufer, *Dental Practitioner.*

IF any dentist, in the enthusiasm of youth or the zeal of honest and unselfish intention, thinks he has a mission to do good to his profession, and is anxious to get into harness, he may make up his mind that if he succeeds, he will in the long run get more kicks than kindness. Some people deserve to be kicked. They are intensely for self and the dollar, and if they are not actually hypocritical thieves, they run so close to the business of stealing that they only owe their escape to their talent for concealment But the most of hard workers in official capacities in our ranks are incorruptible and above reproach. In fact they are as they should be, more scrupulous of funds which do not belong to them, than of that which is their own. We find rascality suspected and proved in every sphere of life. Dentists are not demigods; but, as a rule, they are very respectable citizens who rarely. if ever, get into the penitentiary. They are not solicitous of public office, because they have not the time. Yet there are some who give a great deal of time and thought to the public service and receive public gratitude. The odd thing is that when they serve the profession in the same way they provoke professional jealousy. Somebody must serve the profession. There are ambitious cranks, and very respectable, but wholly unquaified men, who hunger to serve in any and every official capacity. But happy after all is the man who minds his own business.—Dominion Dental Journal.

---

TO FOLD GOLD FOIL WITHOUT CONTACT OF THE FINGERS.—The book of foil is held in the left hand and opened to the first leaf, the right half of the book is slightly raised so that the outer edge of the sheet of foil nearest the right hand will fall, with the help of a little shaking, inward so that the two edges will be approximated at the central fold of the book, which is then closed on the folded gold and pressed into contact with the fingers passed over the outside covers of the book. The same process is repeated until the leaf of foil is folded to the desired number of thickness. The strip of folded foil can then be cut into strips the desired width with shears.—*Dental Brief*.

MATRICES.—At a recent meeting of the Chicago Dental Society the question of using matrices was discussed to a slight extent by some of the members. Surprise was expressed by one gentleman that an old practitioner had adopted the matrix at this late day. Matrices have been used in filling teeth for many years, especially when using the plastics. In regard to the use of the matrix in gold filling there is still a division of opinion. Some contend that a matrix may prevent the perfect joint on the long axis of a tooth, but we believe that a little careful practice will soon make the indifferent operator a more careful and painstaking worker. The matrix reduces to a minimum the subsequent polishing of the filling, and if a matrix is carefully and firmly adjusted, the contour is more easily made than without its use. The interproximate space is always better protected by the use of a matrix at an expense of less labor for the operator. We are in favor of matrices as time-savers and as useful adjuncts in contouring of molars and bicuspids with gold, tin and amalgam, as well as the various cements.—Dental Review.

---

TREATMENT FOR EXPOSED DENTIN.—Sometimes, when much absorption of the gum has taken place, the tooth becomes very sensitive to changes of temperature. The following method has lately been recommended to guard the tooth from thermal changes. A good plate is fitted over the exposed fangs, a model being first taken, which is pared away at the neck of the teeth so that a band would fit beneath the gum. Chloro percha is painted beneath the gum and the plate fastened with a pin and cemented the same as with a crown. The relief has been found immediate, though before the plate was put on the patient could not bear either hot or cold water in the mouth. The tooth has remained quite well, and is doing good service. In another case the symptoms were the same, but complicated by decay on the distal surface. The cavity was filled with artificial dentin, then a split band was fitted around the tooth, which was tightened up by a screw when in position. This case has proved equally successful.—*British Journal.*

HARDER THAN A DIAMOND.—Within a few days, according to the mineral collector, the Patent Office will grant title in a discovery which may fairly be considered as being the most remarkable since the X-ray. It is for a substance that is harder than the diamond, and the inventor is Moissan, the French savant, whose experiments in the line of diamond-making by artifice have obtained such wide publicity. The utmost secrecy has been maintained in regard to the matter, but investigation reveals the fact that the substance in question is a carbide of titanium; that is to say, a compound of carbon with the metal titanium. There can be no doubt that its production in quantities will revolutionize many industries where abrasives are employed, and it may even be used for the cutting of diamonds.

Titanium is one of the most interesting of the rare metals. It is about half as heavy as iron, and, like the latter, it is white when perfectly pure. Chemically, it resembles tin, while in its physical proportions it is like iron. The familiar mineral "rutile" is an oxide of titanium, and is used to give the proper color to artificial teeth. A small quantity of the mineral put into the mixture for tooth enamel produces the peculiar yellowish tint that counterfeits nature so admirably.—Journal of Medicine and Science.

---

A NEW ARMY HOSPITAL.—The largest under the control of the Government, is to be constructed of Georgia pine, at Savannah, Georgia, at a cost of $150,000. There will be 149 buildings, all designed and constructed with a view to permanency. The ground plan of the buildings will be rectangular, with covered ways connecting all the buildings with each other and with the administration building in the center. Each of the four sides of the quadrangle will contain a ward with a capacity of 250 beds. One ward will be devoted to surgical cases, and an enclosed passageway will connect it with a modern operating-room. In addition to the general wards, there will be private rooms for the accommodation of invalid officers. An unlimited supply of artesian water will be provided. Coal base-burners will supply heat. An ice factory is situated within fifty yards of the site, and the cost of ice is inconsiderable. The officers' quarters will be of two stories. The dormitories for the nurses and the hospital corps will occupy two buildings. The chief surgeon will have a private residence.—Atlanta Medical and Surgical Journal.

---

IT IS a fine thing to have the reputation of being a fast workman, but it is of much more importance to be known as a good workman. If every detail is done accurately, the fingers will soon learn speed; but if there is hurry and slight, the work will soon show folly. First accuracy, then speed.

Some writers are like a minister I once had an appointment with at a country schoolhouse. He spoke first and I was to catch inspirations from what he might say. He floundered about for quite a time saying nothing and getting nowhere.

As we were walking home I said to him, "What were you trying to get at for the first fifteen minutes of your speech ?"

"An idea," he replied, "and it took fifteen minutes to catch it."

So with many writers. They take their pen in hand and scribble away for an idea, and it sometimes takes fifteen minutes to catch it.

In reading an exhaustive essay we often have to read much before reaching the real meat of the subject.

Oh, for condensation !—*Dental Brief.*

---

IN attempting to extract an upper left first bicuspid root a prominent dentist recently met with an accident previously unheard of by me. The third molar and second bicuspid were in situ, the first and second molars had been rapidly reabsorbed. The roots of the first bicuspid had been broken off high up above the gingivæ, and in attempting to force it out the entire alveolar process surrounding both bicuspids was fractures.—*Dental Century.*

DR. BLACK'S THEORY.—Dr Black says that all teeth are equally soft; that the dentine of all teeth is the same in density; that they have the same specific gravity; that a good tooth is no better than a bad tooth; that any filling-material that will save one tooth will save any other, and that there is no choice except on the part of the dentist between lead or tin or gold or amalgam. Well, now, I do not believe that bad teeth are always bad. I do not believe that teeth that are bad in the beginning are bad all through life. I am honest in being mistaken, if I am mistaken, but I believe I have seen changes in the teeth of some of my patients. I call to mind a patient who for ten years suffered from caries of the teeth. He was then leading a sedentary life. He changed his occupation, becoming a railroad man, thus securing plenty of air and exercise. His teeth before required constant care. Now he will go sometimes for five or more years without a single cavity in his teeth. He is an unprofitable patient because his teeth do not require any care. Dr. Black would say that they could not change for the better.—Dr. Edwin T. Darby.

---

SUN baths are almost as essential as water baths, quite as much so for many invalids, and the weak, sallow, spiritless class that are neither sick nor well.

Take the clothing off and sit or lie in a room where the sun shine will come on you. Change your position so as to have its effect on all portions of the body. Never mind the tanning, nor the browning, nor even the reddening, but toughen yourself to its full effect. You will need less clothing after a while, catch cold less easily, and have a more vigorous circulation, better spirits and more strength.—*Dental Brief.*

---

STERILIZING INSTRUMENTS.—Dipping in alcohol and burning off the alcohol effectually destroys all virus or germs. The heat reaches the whole surface of the instrument.—Faneuil D. Weisse, *Dental Cosmos.*

The use of ice in small quantities, frequently repeated, is very general in many diseases, but it is found difficult to keep it from melting, especially when in small blocks. To keep it from melting the ice should be put in a vessel and covered with a plate, and the vessel should be placed on a feather-bed, and covered with a feather pillow or cushion, feathers being almost non-conductors of heat. By this plan a few pounds of ice can he kept for several days, even in summer heat.—*Self-Culture.*

---

SOLDERING GOLD CROWNS.—Make a saturated solution of borax by boiling until no more will dissolve. When the solder is wanted to flow, moisten with this solution, and it will run like a flash. Mix yellow ochre to a creamy consistency for painting the parts to be protected from solder.—John T. Usher, *Dental Cosmos.*

---

EUCAINE.—Eucaine provokes hyperemia at the point of application, and should be used with caution when hemorrhage is to be feared. Five minims of a 10 per cent. solution are sufficient to inject into the gum for the painless extraction of teeth.—Wolff, *Dental Digest.*

---

DENTAL FEES must often take the same course as a basis of settlement as the physician's fees. Discrimination is constant and necessary on account of the fact that all are subject to dental disease and only a small portion of the people are rich.

